# Hypopressive physiotherapy for refractory non-neurogenic underactive bladder in children: a retrospective case series

**DOI:** 10.3389/fped.2026.1879637

**Published:** 2026-07-29

**Authors:** Iván Somoza Argibay, Miriam García González, Silvia Seoane Rodríguez, Laura Vázquez Montes, Samuel González-Pola Yuncal, María Dolores González Castro, Santiago Altamirano, Verónica Montes Villar, Mónica Menéndez Pardiñas

**Affiliations:** 1 Department of Pediatric Surgery, Grupo Emergente de Investigación en Ingeniería Biomédica, Instituto de Investigación Biomédica de A Coruña (INIBIC), Hospital Universitario A Coruña, A Coruña, Spain; 2 Department of Pediatric Surgery, Hospital Universitario A Coruña, A Coruña, Spain; 3 Pediatric Rehabilitation Unit (Physical Medicine and Rehabilitation), Hospital Universitario A Coruña, A Coruña, Spain; 4 Pediatric Rehabilitation Unit (Physiotherapy), Hospital Universitario A Coruña, A Coruña, Spain; 5 Physiotherapy, Medicine and Biomedical Sciences Department, University of A Coruña, A Coruña, Spain

**Keywords:** hypopressive exercises, pediatric urology, posterior urethral valves, post-void residual, underactive bladder, urotherapy

## Abstract

**Background:**

Pediatric non-neurogenic underactive bladder (UAB) is characterized by reduced detrusor contractility, elevated post-void residual (PVR), and recurrent urinary tract infections (UTIs). Standard urotherapy and pelvic floor rehabilitation constitute first-line management, whereas clean intermittent catheterization (CIC) is frequently required in refractory cases. Hypopressive physiotherapy has shown benefit in adult pelvic floor dysfunction but has not been systematically evaluated in pediatric UAB.

**Objective:**

To evaluate the clinical effectiveness of a structured hypopressive physiotherapy program as a specific urotherapy intervention in children with refractory non-neurogenic UAB.

**Methods:**

A retrospective case series including six children with refractory non-neurogenic underactive bladder and baseline post-void residual volume >50 mL despite previous conservative treatment. All underwent a structured hypopressive physiotherapy program. Post-void residual volume, urinary tract infections, clean intermittent catheterization requirement, and uroflowmetry findings were evaluated at baseline, 6 months, and 12 months. Paired comparisons were performed using the Wilcoxon signed-rank test.

**Results:**

Six children were included, including two with bladder dysfunction secondary to previously treated congenital lower urinary tract obstruction (posterior urethral valves and ureterocele, respectively). Mean baseline PVR was 123.3 ± 20.5 mL. At 6 months, mean PVR decreased to 29.2 ± 16.9 mL (*p* = 0.031). At 12 months, mean PVR was 12.5 ± 9.9 mL (*p* = 0.028). All patients achieved PVR ≤20 mL during follow-up. Recurrent UTIs resolved in all cases. One patient discontinued CIC. Uroflowmetry curves partially normalized in three patients, while two maintained plateau morphology despite marked PVR reduction.

**Conclusions:**

In this preliminary retrospective case series, hypopressive physiotherapy was associated with substantial reductions in post-void residual volume in this small cohort. These findings from this preliminary case series support further prospective controlled studies.

## Introduction

Lower urinary tract dysfunction (LUTD) is among the most frequent conditions encountered in pediatric urology practice (1–3). Standard urotherapy—including education, behavioral modification, hydration optimization, bowel management, and scheduled voiding—constitutes first-line treatment and is effective for many functional voiding disorders (1–4). However, a subset of patients evolves toward chronic inefficient bladder emptying characterized by elevated post-void residual (PVR), recurrent urinary tract infections (UTIs), and progressive bladder dysfunction (4).

Underactive bladder (UAB) in children is defined by reduced detrusor contractility resulting in incomplete bladder emptying and increased residual urine (5). Pediatric UAB may develop as a late stage of longstanding LUTD or in association with congenital lower urinary tract obstruction (LUTO), such as posterior urethral valves (PUV), even after relief of obstruction (6, 7, 14). Management options are limited and typically include double voiding, pelvic floor rehabilitation or biofeedback (4–7). Pharmacological treatment options for pediatric underactive bladder remain limited. Cholinergic agonists such as bethanechol have historically been used in an attempt to improve detrusor contractility, although evidence supporting their efficacy is weak and inconsistent. Consequently, most contemporary management strategies rely on behavioral interventions, pelvic floor rehabilitation, and clean intermittent catheterization rather than pharmacological stimulation of bladder contraction (5–7).

Although biofeedback has demonstrated efficacy in dysfunctional voiding (7, 12, 13), evidence for specific conservative treatments targeting pediatric detrusor underactivity remains scarce. CIC is effective but may impose psychological burden and long-term dependence, particularly in adolescents.

Hypopressive physiotherapy comprises posture- and breathing-based exercises intended to reduce intra-abdominal pressure and modulate activation and coordination of deep abdominal and pelvic floor musculature (8, 9). In adult populations, randomized controlled trials have reported improvements in pelvic floor function and urinary symptoms (8, 9), although systematic reviews highlight heterogeneity and limitations in the evidence base (10, 11). To date, pediatric studies evaluating hypopressive physiotherapy for UAB are lacking. Unlike conventional biofeedback, which requires active interpretation of visual or auditory feedback signals, hypopressive physiotherapy is based on guided postural and breathing exercises that can be adapted to the developmental level of pediatric patients. The technique is non-invasive, does not require specialized equipment during home practice, and may facilitate patient engagement through structured physical activity. For these reasons, it may represent an attractive adjunctive rehabilitation strategy in children and adolescents with chronic voiding dysfunction.

This study aimed to evaluate preliminary clinical evidence of hypopressive physiotherapy as a specific urotherapy intervention in children with refractory non-neurogenic UAB.

## Materials and methods

### Study design

A retrospective case series was conducted at Hospital Universitario A Coruña.

### Participants

Six children with non-neurogenic UAB and baseline PVR >50 mL were included. Adequate sphincter relaxation during voiding was confirmed in all patients based on uroflowmetry and pelvic floor assessment, thereby excluding dysfunctional voiding. Diagnosis of non-neurogenic underactive bladder was established based on persistent elevated post-void residual volume (>50 mL), symptoms of incomplete bladder emptying, exclusion of neurological and active anatomical causes, and non-invasive functional assessment. Two patients had bladder dysfunction secondary to previous congenital lower urinary tract obstruction, caused by posterior urethral valves in one case and obstructive ureterocele in the other. No patient had active obstruction at inclusion. Urodynamic studies were available in four of the six patients. The remaining two patients were diagnosed based on clinical findings, ultrasound assessment, uroflowmetry, and exclusion of neurological or anatomical causes. The two patients with previous congenital lower urinary tract obstruction underwent videourodynamic evaluation, which confirmed detrusor underactivity after relief of the obstruction. Individual clinical characteristics, previous management, and diagnostic evaluation are summarized in Table 1.

**Table 1:** Individual clinical characteristics, clinical background, diagnostic evaluation, and outcomes of the six children included in this retrospective case series.

Patient	Age (y)	Etiology (LUTO)	Clinical background and diagnostic evaluation	Urodynamics	Baseline PVR (mL)	6-mo PVR	12-mo PVR	Baseline UTIs	CIC	Baseline uroflow	Final uroflow
1	12	No	Idiopathic refractory UAB with recurrent cystitis despite standard urotherapy and biofeedback. Urodynamic study demonstrated detrusor underactivity. Required CIC before physiotherapy.	UDS	150	20	0	2 cystitis	Yes	Flat/plateau	Plateau, low Qmax
2	14	No	Idiopathic refractory UAB with recurrent cystitis despite standard conservative management.	Not performed	100	40	20	3 cystitis	No	Interrupted	Interrupted
3	12	Yes (PUV)	History of posterior urethral valves treated in infancy. Persistent incomplete bladder emptying after successful valve ablation. Videourodynamic study confirmed detrusor underactivity in the absence of persistent outlet obstruction.	VUDS	100	60	20	1 PN/2 cystitis	No	Flat/plateau	Bell-shaped
4	14	No	Idiopathic refractory UAB with recurrent pyelonephritis and cystitis. Urodynamic study demonstrated detrusor underactivity.	UDS	140	20	20	2 cystitis/1 PN	No	Interrupted	Bell-shaped
5	10	No	Idiopathic refractory UAB with persistent elevated post-void residual despite standard urotherapy.	Not performed	140	20	0	None	No	Plateau	Bell-shaped
6	12	Yes (Ureterocele)	History of obstructive ureterocele treated surgically. Persistent bladder emptying dysfunction after relief of obstruction. Videourodynamic study confirmed detrusor underactivity.	VUDS	110	15	15	None	No	Plateau	Plateau

CIC, clean intermittent catheterization; LUTO, lower urinary tract obstruction; PN, pyelonephritis; PUV, posterior urethral valves; PVR, post-void residual; UAB, underactive bladder; UDS, urodynamic study; VUDS, videourodynamic study.

**Inclusion criteria:** (i) diagnosis of non-neurogenic UAB; (ii) baseline PVR >50 mL; (iii) failure of standard urotherapy and/or biofeedback; (iv) minimum follow-up of 6 months.

**Exclusion criteria:** (i) neurogenic bladder; (ii) active anatomical obstruction; (iii) simultaneous introduction of other treatments during follow-up.

### Outcome measures

Post-void residual volume (PVR) by ultrasound, UTI recurrence rate, need for CIC, and uroflowmetry morphology were evaluated at baseline, 3–6 months, and 12 months. PVR was measured by ultrasound immediately after voiding.

#### Hypopressive physiotherapy protocol

##### Treatment schedule

The program was delivered by a physiotherapist specialized in pediatric pelvic floor rehabilitation. The protocol included postural alignment, diaphragmatic breathing, abdominal vacuum techniques, and progressive neuromuscular activation. Sessions were performed in a supervised and standardized manner using four predefined postures. Exercises were conducted slowly and under continuous guidance to ensure correct technique. Each exercise was repeated three times in each posture. Sessions were performed weekly for three months and monthly thereafter. Following initial instruction and supervised training, patients were encouraged to perform the exercises daily at home throughout the study period. No additional new therapeutic interventions were introduced during follow-up. Compliance with home exercises was assessed during follow-up visits through patient and family reporting.

##### Exercise technique

The hypopressive maneuver consisted of a complete expiration followed by an end-expiratory apnea. During apnea, patients performed a simulated inspiratory movement without allowing air entry (inspiratory aspiration maneuver through voluntary contraction of the serratus anterior and the rib cage elevator muscles), combined with active lateral traction of the humeri and shoulder girdle decoaptation. Each posture was accompanied by axial self-elongation, cervical elongation with chin tuck, glenohumeral decoaptation, anterior displacement of the center of gravity, and maintenance of expiratory apnea for approximately 8–12 s, depending on patient age and tolerance.

##### Physiological rationale

This maneuver promotes thoracic expansion while reducing intrathoracic pressure, generating a cranial displacement of the diaphragm and a reduction in intra-abdominal pressure. As a consequence, abdominal viscera are displaced cranially. In addition, reflex activation of the deep stabilizing musculature is induced, including the transvs. abdominis, internal oblique muscles, multifidus muscles, pelvic floor musculature, and diaphragm during its relaxation phase.

##### Quality control

**Figure 1:**
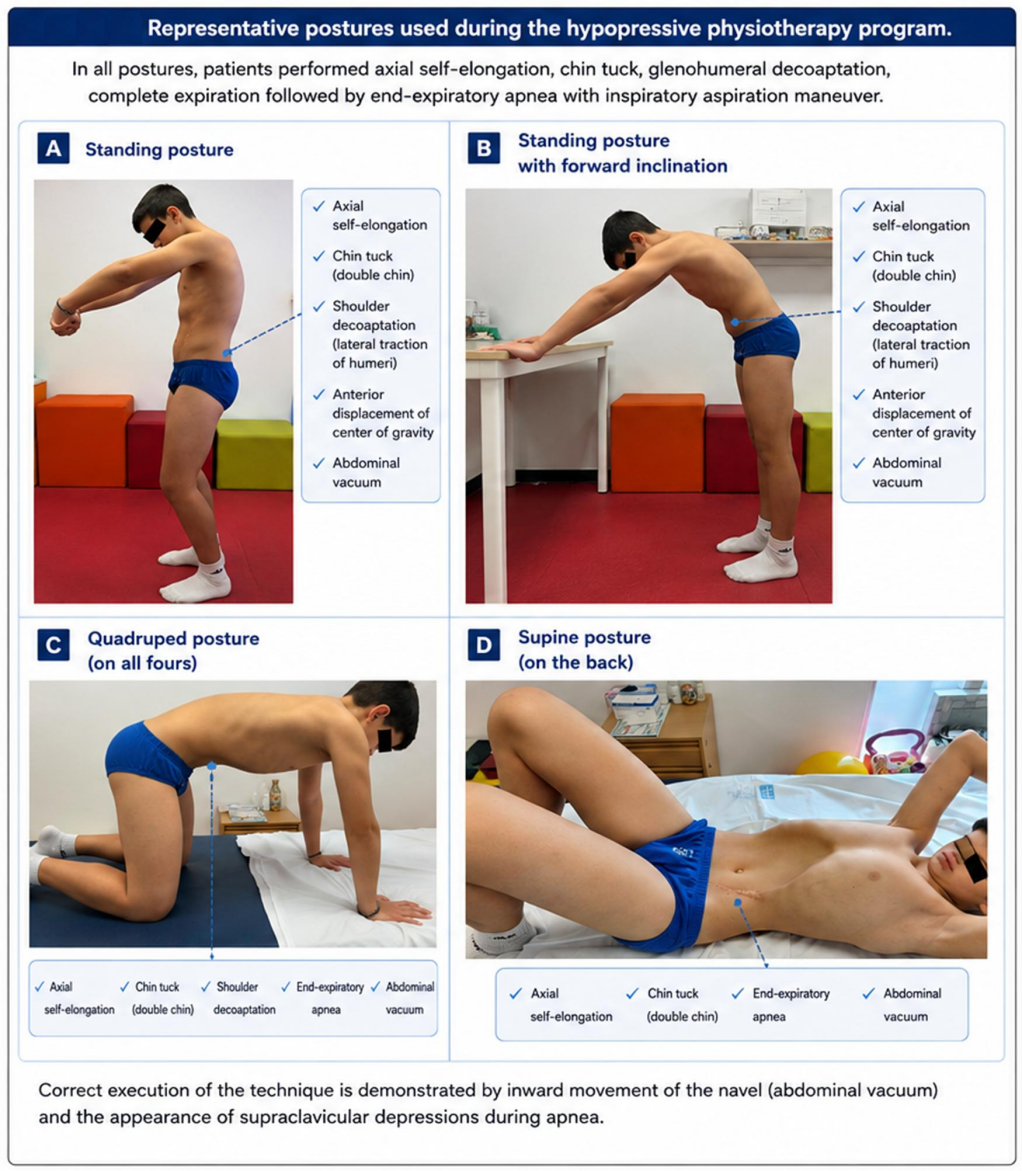
Representative postures used during the hypopressive physiotherapy program. In all postures, patients performed axial self-elongation, chin tuck, glenohumeral decoaptation, complete expiration followed by end-expiratory apnea with inspiratory aspiration maneuver (abdominal vacuum). Correct execution was verified by inward displacement of the umbilical region and supraclavicular depressions during apnea.

**Figure 2:**
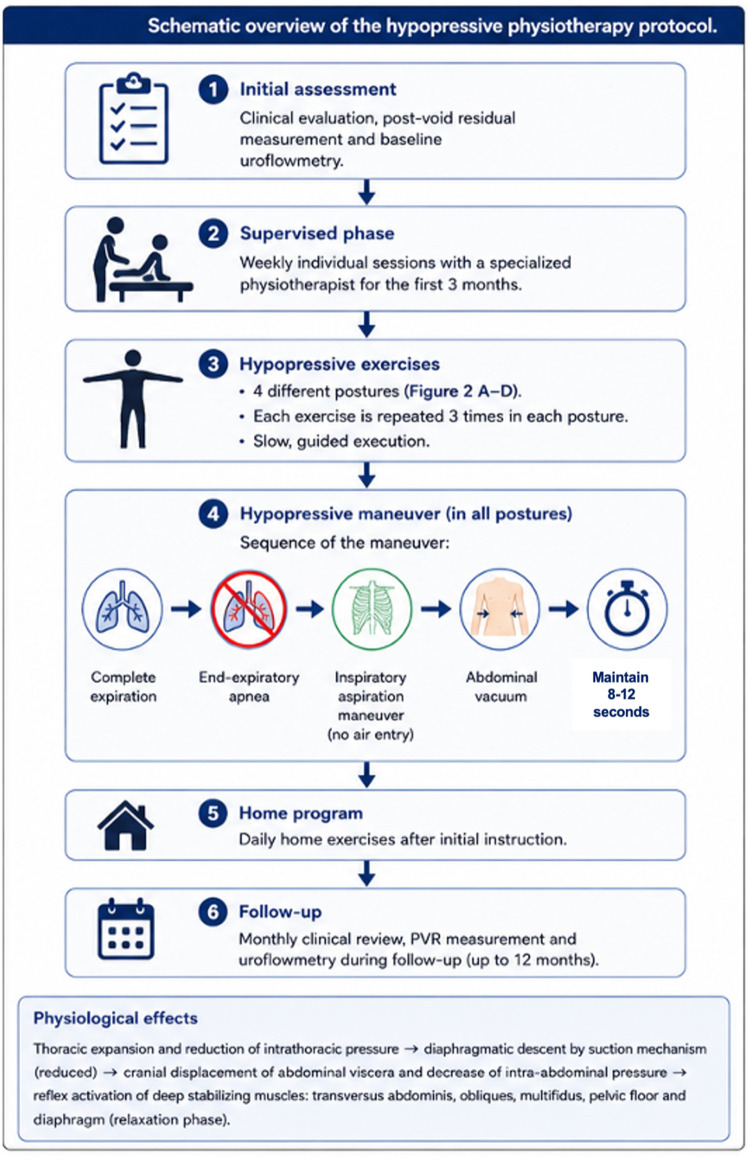
Schematic overview of the structured hypopressive physiotherapy protocol used in this study, including the supervised treatment schedule, home exercise program, follow-up timeline, and sequence of the hypopressive maneuver performed during each session.

Correct execution of the technique was verified by visible inward displacement of the umbilical region (abdominal vacuum) and the appearance of supraclavicular depressions during apnea. Representative postures used during the program are shown in Figure 1, while the overall treatment protocol is summarized in Figure 2.

### Statistical analysis

Continuous variables were presented as mean ± standard deviation (SD). Paired comparisons between baseline and follow-up were performed using the Wilcoxon signed-rank test due to small sample size. A *p*-value <0.05 was considered statistically significant.

## Results

### Patient characteristics

Six children (mean age 12.3 ± 1.6 years) were included. Two patients had bladder dysfunction secondary to congenital lower urinary tract obstruction; they underwent videourodynamic studies, which confirmed impaired bladder emptying secondary to detrusor underactivity in the absence of persistent outlet obstruction. Baseline demographic characteristics, clinical background, and individual patient outcomes are summarized in Table 1.

### Post-void residual volume

The mean baseline PVR was 123.3 ± 20.5 mL.

At 6 months, mean PVR value decreased to 29.2 ± 16.9 mL, representing a 76% reduction from baseline (Wilcoxon *p* = 0.031).

**Figure 3:**
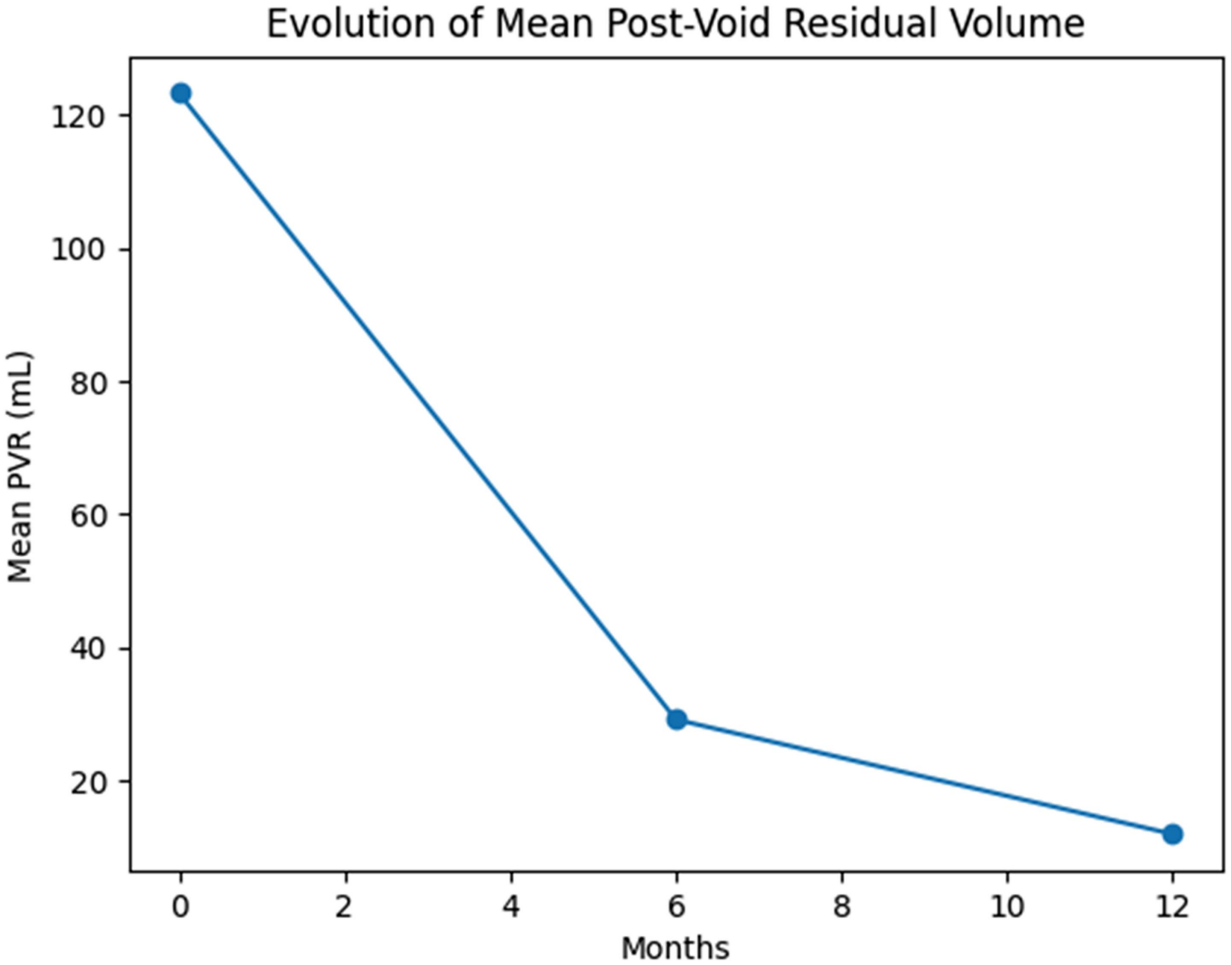
Evolution of mean post-void residual volume (PVR) measured by bladder ultrasound during the 12-month follow-up after initiation of the hypopressive physiotherapy program. Values represent mean ± standard deviation.

At 12 months, mean PVR value was 12.5 ± 9.9 mL, corresponding to a 90% reduction from baseline (Wilcoxon *p* = 0.028). All six patients achieved PVR ≤20 mL at both follow-up intervals. The evolution of mean PVR is illustrated in Figure 3.

### Urinary tract infections and CIC

Four patients (66.6%) experienced recurrent UTIs at baseline (mean 2.2/year). No recurrent infections were documented during follow-up. One patient discontinued clean intermittent catheterization within six months.

### Uroflowmetry findings

Baseline uroflowmetry demonstrated plateau or flattened patterns in all cases. Three patients showed partial normalization toward a bell-shaped curve, while two—including one post-LUTO case—maintained plateau morphology despite normalization of residual volume. Additional quantitative uroflowmetry analysis demonstrated improved voiding dynamics in these patients. In one patient, flow time decreased from approximately 3 min to 1 min, with an increase in Qmax from 7 to 8 mL/s. In the second patient, flow time remained approximately 1 min, while Qmax increased from 9 to 10 mL/s.

## Discussion

Pediatric non-neurogenic underactive bladder (UAB) remains a complex and insufficiently standardized condition within the spectrum of LUTD. ICCS recommendations emphasize standard urotherapy as first-line management, including behavioral modification, hydration optimization, bowel management, and timed voiding (1, 2). While these measures are effective in many functional disorders, specific conservative strategies targeting detrusor hypocontractility are limited. In refractory cases with persistently elevated PVR and recurrent UTIs, CIC often becomes the only reliable means to ensure adequate bladder emptying.

Underactive bladder in children is frequently conceptualized as a late-stage manifestation of longstanding LUTD, sometimes evolving from dysfunctional voiding patterns, infrequent voiding, or maladaptive pelvic floor behaviors (5). In addition, bladder dysfunction secondary to congenital LUTO, such as PUV, may persist despite early anatomical correction. Longitudinal studies in PUV populations have described progressive detrusor remodeling and hypocontractility, resulting in inefficient emptying and chronic residual urine despite early valve ablation (6, 7). These children remain at risk of recurrent infection and potential upper tract involvement.

In this cohort, hypopressive physiotherapy was associated with consistent reductions in PVR across all six patients, including two with prior LUTO due to PUV and ureterocele, respectively. The magnitude of improvement—76% at six months and 90% at twelve months—suggests a clinically meaningful effect despite the small cohort size.

The inclusion of two patients with bladder dysfunction secondary to congenital LUTO enhances clinical relevance. In both patients, videourodynamic evaluation confirmed detrusor underactivity after relief of the obstruction, supporting their inclusion within the spectrum of underactive bladder rather than persistent outlet obstruction. Although etiologies differed, all patients shared persistent incomplete bladder emptying despite previous standard management, supporting their inclusion within the same clinical phenotype. Bladder remodeling and progressive detrusor hypocontractility are well-recognized sequelae of posterior urethral valves, often resulting in persistent emptying dysfunction despite early anatomical correction. The improvement observed in these cases suggests that modulation of abdominal–pelvic coordination and pressure dynamics may enhance voiding efficiency independently of intrinsic detrusor recovery.

An important finding was the discordance between improvement in PVR and incomplete normalization of uroflowmetry morphology in some patients. Two patients maintained plateau-shaped uroflowmetry patterns despite marked reductions in post-void residual volume. Additional quantitative uroflowmetry analysis demonstrated improvement in voiding dynamics. In one patient, flow time decreased from approximately 3 min to 1 min, with an increase in Qmax from 7 to 8 mL/s. In the second patient, flow time remained approximately 1 min, while Qmax increased from 9 to 10 mL/s. These findings suggest that improvements in bladder emptying and voiding dynamics may occur despite persistence of abnormal uroflowmetry morphology. A plausible interpretation is that hypopressive training may improve abdominal–pelvic coordination and pressure modulation during voiding without directly restoring intrinsic detrusor contractility.

Hypopressive physiotherapy is based on postural control, diaphragmatic breathing, and abdominal vacuum techniques designed to reduce intra-abdominal pressure and modulate activation of deep abdominal and pelvic floor musculature (8, 9). Evidence in urology has primarily been generated in adult women with pelvic floor dysfunction and urinary incontinence. Randomized trials have reported improvements in pelvic floor activation and urinary symptoms following structured hypopressive programs (8, 9). However, systematic reviews emphasize heterogeneity of protocols and limited methodological robustness (10, 11). Importantly, adult studies focus predominantly on stress urinary incontinence rather than detrusor underactivity, and pediatric evidence is lacking.

The mechanisms underlying the observed improvements remain uncertain. Several hypotheses may be considered: enhanced pelvic floor relaxation and coordination during voiding; optimization of intra-abdominal pressure timing and magnitude; and neuromuscular re-education supporting central patterning of coordinated abdominal–pelvic synergy. These hypotheses warrant evaluation in prospective studies incorporating objective mechanistic outcomes such as urodynamic testing, symptom scores, and quality-of-life measures.

To our knowledge, this is the first report describing hypopressive physiotherapy as a specific urotherapy intervention in pediatric non-neurogenic underactive bladder. Although pediatric evidence remains absent in the literature, adult data on hypopressive physiotherapy in pelvic floor dysfunction provide indirect biological plausibility. Prospective controlled multicenter studies are required to confirm efficacy, clarify mechanisms, and define optimal patient selection criteria.

This study has limitations, including retrospective design, small sample size, and absence of a control group. Spontaneous maturation, behavioral adaptation, and regression to the mean cannot be excluded. Although urodynamic studies were available in four patients, they were not performed systematically in the entire cohort because of the retrospective study design. Nonetheless, the consistency of response across a heterogeneous cohort—including post-LUTO cases—supports further investigation. Prospective controlled multicenter trials comparing hypopressive physiotherapy with standard urotherapy and biofeedback would clarify efficacy, define optimal protocols, and identify pediatric phenotypes most likely to benefit.

## Conclusions

This preliminary case series suggests that hypopressive physiotherapy may be associated with clinically meaningful reductions in post-void residual volume and resolution of recurrent urinary tract infections in this small cohort of children with refractory underactive bladder. Given the retrospective design, limited sample size, and absence of a control group, these findings should be considered preliminary and hypothesis-generating. These findings support further prospective controlled studies to evaluate efficacy and clarify mechanisms of action.

## Data Availability

The original contributions presented in the study are included in the article/Supplementary Material, further inquiries can be directed to the corresponding author/s.
